# Cephalopod palaeobiology: evolution and life history of the most intelligent invertebrates: Honoring Sigurd von Boletzky and his contributions to cephalopod research

**DOI:** 10.1186/s13358-022-00247-1

**Published:** 2022-05-19

**Authors:** Christian Klug, Laure Bonnaud-Ponticelli, Jaruwat Nabhitabhata, Dirk Fuchs, Kenneth De Baets, Ji Cheng, René Hoffmann

**Affiliations:** 1 Paläontologisches Institut Und Museum, Universität Zürich, Karl-Schmid-Strasse 4, 8006 Zürich, Switzerland; 2 Lab BOREA MNHN/CNRS/SU/UCN/IRD/UA, 43 rue Cuvier-CP26, 75005 Paris, France; 3 Excellence Centre for Biodiversity of Peninsular Thailand (CBIPT), Faculty of Science, Prince of Songkla University, Hatyai, 90112 Songkhla Thailand; 4 SNSB-Bayerische Staatssammlung Für Paläontologie Und Geologie, Richard-Wagner-Straße 10, 80333 Munich, Germany; 5 GeoZentrum Nordbayern, Fachgruppe PaläoUmwelt, Friedrich-Alexander-University Erlangen-Nürnberg, Loewenichstr. 28, 91054 Erlangen, Germany; 6 State Key Laboratory of Palaeobiology and Stratigraphy, Nanjing Institute of Geology and Palaeontology and Center for Excellence in Life and Paleoenvironment, Chinese Academy of Sciences, 39 East Beijing Road, Nanjing, 210008 China; 7 Institute of Geology, Mineralogy, & Geophysics, Ruhr-Universität Bochum, 44801 Bochum, Germany

**Keywords:** Coleoidea, Ammonoidea, Nautilida, Anatomy, Embryology, Actualism

## Abstract

Sigurd von Boletzky was a cephalopod researcher who was world-renowned for his enthusiasm for his field of research, for his friendly and calm personality, and, of course, his publications. He dedicated most of his life as active researcher on the development, biology and evolution of coleoids. Nevertheless, he was always curious to learn about other cephalopods as well. Sigurd passed away in Switzerland on September 28th 2020. We dedicate this text and volume to his memory.

## Introduction

**Fig. 1:**
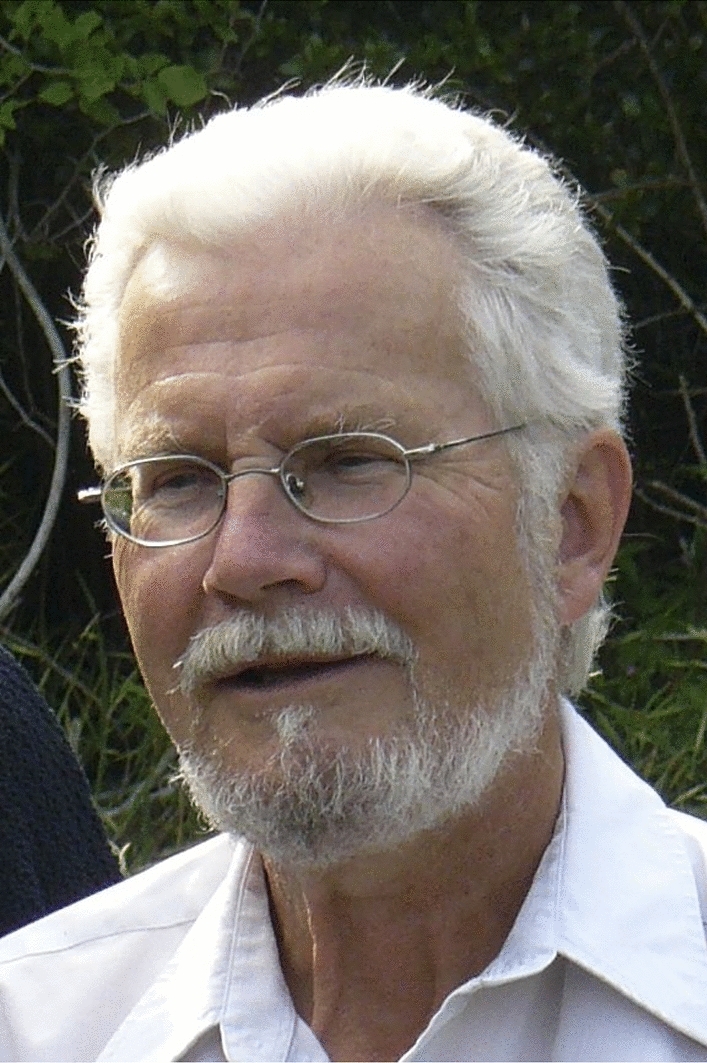
Portrait of Sigurd von Boletzky (photo by courtesy of his wife Verena von Boletzky)

Detailed knowledge, great enthusiasm and a general deep love for coleoid cephalopods characterized the researcher Sigurd von Boletzky (Fig. 1). His personality was nicely portrayed in an online newsletter of the cephalopod International Advisory Council by Rodrigues (2012): “Everyone likes Sigurd. The ladies say he is a gentleman and the gentlemen say the same”. In this editorial, we want to provide a short vita and highlight some of his achievements to commemorate him. Additionally, we attach a hopefully complete bibliography.

This volume includes various contributions on all main groups of cephalopods. Of course, the modern coleoids were most relevant for the life of Sigurd and hence, we included several articles on that topic (Bello & Deickert, 2021; Neige, 2021; Ward et al., 2022; Ziegler et al., 2021). He was similarly interested in coleoid evolution and thus stem group coleoids (Fuchs et al., 2021; Klug et al., 2021b; Nabhitabatha, 2022) including belemnites (De Baets et al., 2021; Jagt-Yazykova et al., 2021; Klug et al., 2021c). Being closely related to coleoids, articles on ammonoid palaeobiology had to be included in the volume as well (Beck et al., 2021; Weber et al., 2022). Ultimately, the iconic nautilids cannot miss and are represented by two articles on Cretaceous forms from the Middle East (Klug et al., 2021; Sharifi et al., 2021).

The biographic information provided here is extracted from Rodrigues (2012), the personal CV of Sigurd, as well as letters and texts, which we kindly received from his wife Verena von Boletzky and his colleagues Jürg Marthy, Giambattista Bello, Nicole Coineau, as well as Angel Guerra.

## Life history of Sigurd von Boletzky

Although some colleagues perceived him as French or Swiss, his German accent revealed his origin; his native town was Frankfurt am Main, where his embryonal development ended in 1942. His mother was the German Elisabeth Charlotte von Boletzky (maiden name Schmidt), who was married to Gleb von Boletzky, who came from Russia. When he turned seven, his family moved back to Basel, where he and his older brother Nikita attended school. Having accepted Swiss nationality, he had to serve in the Swiss army in 1961. With the Swiss high school diploma in his pocket, he studied biology/ zoology at the University of Basel from 1961 to 1967. After his diploma (= Master in Biology), he continued in 1964 with his PhD-thesis on "Untersuchungen über die Organogenese des Kreislaufsystems von *Octopus vulgaris* Lam.” [“Studies on the organogenesis of the circulatory system of *Octopus vulgaris* Lam.”] (Boletzky, 1968). As part of his PhD, he began research at Banyuls-sur-Mer at the Laboratoire Arago in southwestern France. He was supervised by Professor Adolf Portmann (Basel University).

After his PhD, he carried out a PostDoc in the USA with a scholarship granted by the Swiss National Science Foundation (1968 to 1969) at the Institute of Marine and Atmospheric Sciences, University of Miami FL, and at the Marine Biological Laboratory, Woods Hole MA. At this occasion, he had the opportunity to join a research expedition on the “RV John Elliott Pillsbury”. During this cruise, he received specimens, which he used for his articles (Boletzky 1970b, 1971a; Boletzky and Boletzky, 1973a, 1973b, 1973c) about the two genera *Semirossia* and *Neorossia* (Subfamily Rossiinae). 1968 was a good year, because in addition to the publication of his thesis, he married Maria Verena Lötscher, a certified biomedical assistant. Their daughters Paula Sophia (*1972) and Julia Dominika (*1974) attended school in France and went for higher education in Switzerland.

In 1969, he returned to Banyuls with a Swiss scholarship by the Janggen-Pöhn Foundation. Subsequently, the French National Research Center (C.N.R.S.) (Attaché de recherche 1969–1977) employed him to continue research in cephalopod biology at the Laboratoire Arago, Banyuls-sur-Mer. Colleagues who knew him well agree that he enjoyed his work there. He then completed his inaugural dissertation on the post-embryonic development of cephalopods (Docteur ès Sciences, Doctorat d'Etat) at the Paris University Pierre et Marie Curie (UPMC, Paris VI) in 1975.

He was a civil servant with the CNRS, first as ‘Chargé de recherche’ from 1977 to 1999, then as ‘Directeur de recherche’ until 2007 and after his retirement in July 2007 as ‘Directeur de recherche honoraire au CNRS’.

## Sigurd’s scientific development and network

### Scientific influence

His professor of zoology at the Basel Institute, Prof. Dr. Adolf Portmann (*1897 to †1982), was likely the person who most decisively directed his scientific career including the broad lines of his approaches and thoughts. Through numerous publications, Sigurd shows his admiration and respect for Portmann and his thoughts.

Another personality who influenced Sigurd was Pio Fioroni (*1933 to †2003). During Sigurd’s studies in Basel, Fioroni was Portmann's first assistant. Later, he became professor of zoology at the University of Münster (Germany). He visited Sigurd in Banyuls many times, which allowed Sigurd to have abundant discussions with him.

Having studied in Switzerland, it was notably the influential work (e.g., Naef, 1922) and reflections of Adolf Naef (*1883 to †1949), which led Sigurd towards phylogenetic and evolutionary interpretations of his research results (see also Boletzky, 1999d). The enormous respect Sigurd had for this tireless researcher who died prematurely at the young age of only 57 years is reflected in the English translation of the standard work "Cephalopoda—Embryology", part I, vol. II, Fauna Flora of the Bay of Naples (Teil I: Naef 1923–1928; vol. II: Naef, 1972). Besides Adolf Naef, Pio Fioroni and Sigurd, the interest of the Swiss in cephalopods is also strongly associated with Adolf Portmann, Hans-Jürg Marthy, and Katharina Mangold-Wirz. Adolf Portmann arrived in Banyuls for the first time in 1925.

### How did Sigurd’s appreciation of cephalopods develop?

The cephalopods, especially their mode of development and embryology fascinated Sigurd and he published about 80 articles about these topics (Boletzky, 1966, 1967, 1968, 1969, 1971b, 1971c, 1971d, 1972b, 1973a, 1973b, 1974a, 1974b, 1975b, 1975c, 1975d, 1977b, 1978a, 1978b, 1978c, 1978–1979, 1979a, 1979b, 1979c, 1980a, 1980b, 1981a, 1982a, 1982b, 1982c, 1984, 1986a, 1987e, 1987f, 1987g, 1988a, 1989b, 1989a, 1989b, 1991, 1992a, 1992b, 1994, 1996a, 1996b, 1997b, 1999b, 2000a, b, 2003a, 2006, 2009a, 2009b, 2010; Boletzky et al., 1970a, 1970b, 1973a, 1973b, 1989, 2001, 2002, 2006; Boletzky and Boletzky, 1973a, 1973b, 1973c; Bandel & Boletzky, 1979, 1988; Boletzky & Fioroni, 1990; Boletzky & Mangold, 1989; Boletzky & Wiedmann, 1978; Bonnaud-Ponticelli & Boletzky, 2016; Chirat & Boletzky, 2003; Fioroni & Boletzky, 1990; Overath & Boletzky, 1974; Mangold & Boletzky, 1973; Mangold et al., 1971; Poulin et al., 2001; Shigeno et al., 2010).

He mainly investigated the early stages of ontogenesis, embryonic development and early juvenile stages, as well as reproduction. For example, it was him who discovered the function of the Kölliker organ as a gland for the hatching of eggs (Boletzky, 1973b). As well, it was also him who described the important relationship between relative egg size and the mode of life of the hatchlings (cephalopod egg size varies from < 2 mm to several centimetres) and the relationship between temperature and development time of the eggs (e.g., Boletzky, 1986b, 1988a, 1994a, 2003a). And it was again Sigurd who described the fractional emission of the eggs by the females in *Sepia officinalis* and therefore in other species (Boletzky, 1972b, 1975d). The comparative study of reproductive strategies followed, of which he was a forerunner.

Sigurd and Hans-Jürg Marthy belonged to the last doctoral students of Portmann who promoted the interest of these two young scientists in the developmental biology of Cephalopods. Consequently, Katharina Mangold-Wirz suggested that, once their theses have been completed, they come and work with her in Banyuls. Therefore, these Portmann students arrived in 1969 and 1970 at the Arago Laboratory in Banyuls and entered the CNRS in 1970 and 1971 after their post-doctoral internships (Sigurd in Miami, USA, and Marthy in Utrecht, Netherlands, and Paris, France).

Sigurd was, according to Marthy, particularly proud of his publication "Our current knowledge of octopod development" [transl. from French] (Boletzky, 1978a, 1978b, 1978c–1979). In fact, he was especially fascinated by the embryology of octopods (especially the phenomenon of reversals of embryos in the chorion and the link of this phenomenon with the mode of hatching). He was also proud of his success in breeding cephalopods, which opened up the possibility of carrying out studies on their post-embryonic and juvenile life (Boletzky, 2004c). Starting in the 1970s, the role of the yolk in embryogenesis was also dear to him (Boletzky, 1975c, 2010).

### Sigurd’s influence outside the field of embryogenesis

Sigurd’s ingenuity becomes also obvious in phylogenetical and palaeontological contributions. His cultivated manners have been proven when he constructively criticized the Neocoleoidea concept, whereupon crown octobrachians and crown decabrachians are sister groups (Boletzky, 1992a, 1992b). His more theoretical contributions (e.g., “Nude ammonites….”, Boletzky, 2004a, 2004b, 2004c; “From head to foot—and back again…”; Boletzky, 2006; “Hatch-as-hatch-can….”, Boletzky, 2007; “Origin of the lower jaw in cephalopods: a biting issue”; Boletzky, 2010) are still stimulating students to critically scrutinize publications.

### Why did he choose developmental cephalopod zoology?

Sigurd is undoubtedly one of the most productive and the more "generalist" of the teuthologists of the second half of the twentieth century. A good part of his research can be tagged as classical descriptive embryology, which brought fourth multiple interpretations, hypotheses and data in systematic, phylogenetic and evolutionary contexts (e.g., Boletzky, , etc.). The common thread in his research was, above all, his fascination with the embryology of cephalopods and developmental biology in genera. In fact, Sigurd is recognized by Rodrigues (2012) as a "developmental and evolutionary biologist".

### Sigurd’s role in the community

Besides his close to 200 publications, he also edited several journals including ‘Vie et Millieu’. When Nicole Coineau took responsibility for the periodical in 1980, Sigurd spontaneously volunteered to help control the English of certain articles when they needed it. He therefore participated effectively in the editorial staff of the periodical more and more regularly as English became the majority language in the journal. He also submitted 18 of his articles for publication in Vie et Milieu (Boletzky, 1972a, 1974c, 1978a, 1978b, 1978c–1979, 1981a, 1984, 1987b, 1997a; Boletzky & Centelles, 1978–1979; Boletzky & Doyle, 1967; Boletzky & Roeleveld, 2000; Boletzky et al., 1997; Boletzky et al., 2002; Boletzky et al., 2006; Bonnaud-Ponticelli & Boletzky, 2016; Hanlon et al., 1985; Kuba et al., 2011; Nishiguchi et al., 2014; Salman et al., 1999). Then, when it was decided in the editorial staff of the periodical to publish themed issues, it organized and edited or co-edited, on various occasions, volumes devoted to Cephalopods alone.

**Fig. 2:**
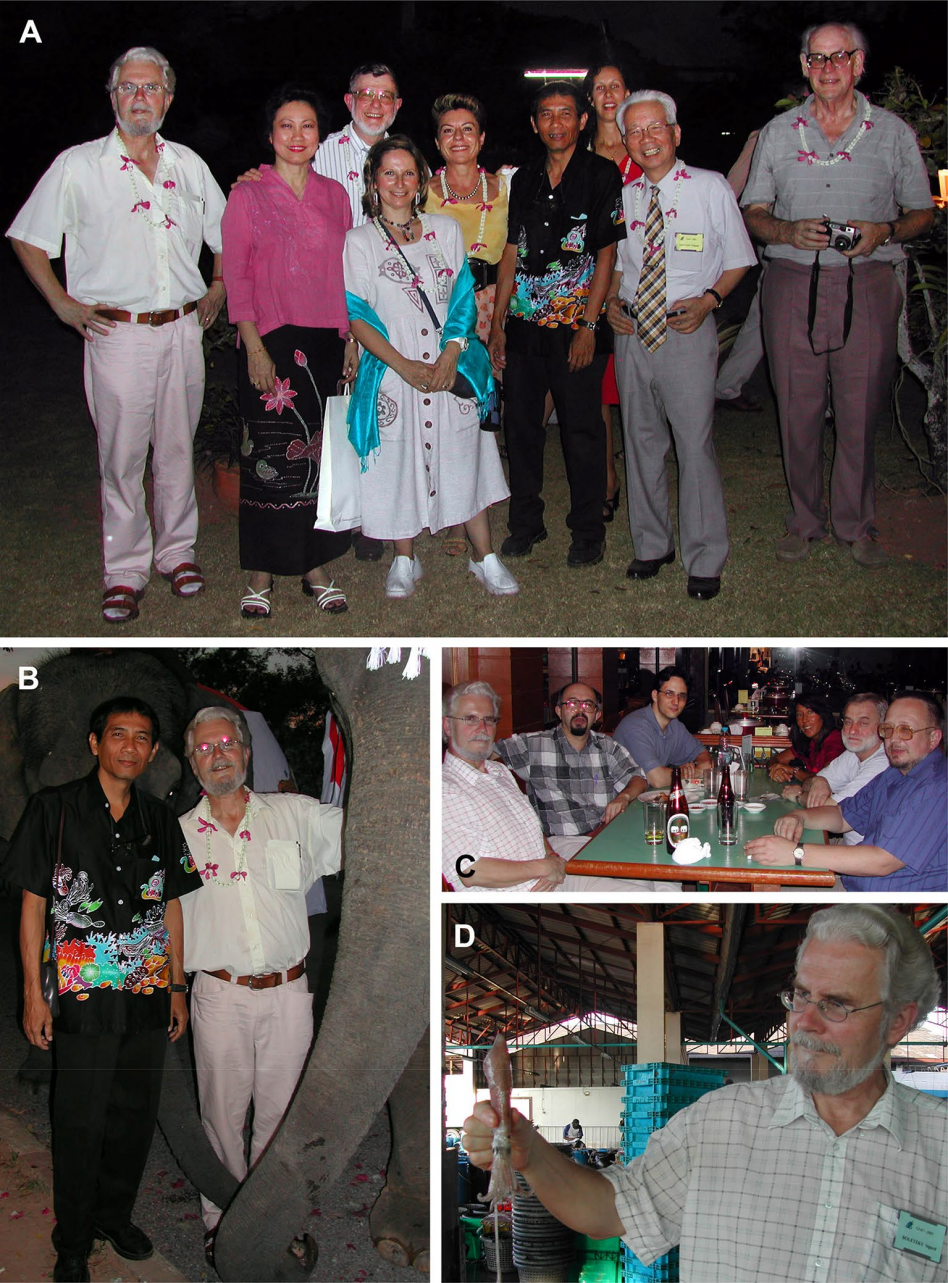
Photos featuring Sigurd von Boletzky at the CIAC meeting 2003 in Phuket, Thailand. A, Group photo at the CIAC2003 Formal Symposium banquet, February 21st, 2003, with Sigurd, Cherdchinda Chotiyaputta, Clyde Roper, Patricia Jereb, Jaruwat Nabhitabhata, Erica Vidal, Takashi Okutani, Malcom Clarke. B, Jaruwat Nabhitabhata with Sigurd and an elephant at the CIAC2003. C, At a restaurant near the Metropole Hotel in Phuket, February 17th, 2003 with Sigurd, Dmitry Alexeyev, Frank E. Anderson, Michele Nishiguchi, Chingis Nigmatulin, Oleg Katigin. D, Sigurd and squid at Phuket fish landing of the Fish Marketing Organization on February 18th, 2003

Most importantly, his enthusiasm, his sense of humour, and his vast knowledge about cephalopod development and evolution is really world-renowned in the community of cephalopod researchers. Notably, we was highly estimated in both the neontological and the palaeontological communities. Therefore, he was a very welcome participant in international scientific meetings. Apart from the regular meetings of the CIAC (Fig. 2), the first of which he co-organized in Banyuls, he organized a number of workshops (reproduction strategies, parasites, sepiolids, systematics, etc.), especially in the Mediterranean region, from which young researchers benefited particularly. His developmental studies have taught researchers a lot about understanding cephalopods, including in the field of palaeontology. Indeed, his work and thoughts on the development and reproduction of cephalopods (Bandel and Boletzky, 1987a, 1987b, 1987c, 1987d, 1987e, 1987f, 1987g; Boletzky 1993, 1997, 2003a; Shigeno et al., 2010) inspired many paleontological studies (Landman et al., 1996; Laptikhovsky et al., 2018) including our own (De Baets et al., 2012).

He also served on the council of the Cephalopod International Advisory Council (CIAC) founded in 1983. The CIAC also made him ‘Honorary life member’ in Vigo, Spain in 2009.

**Fig. 3:**
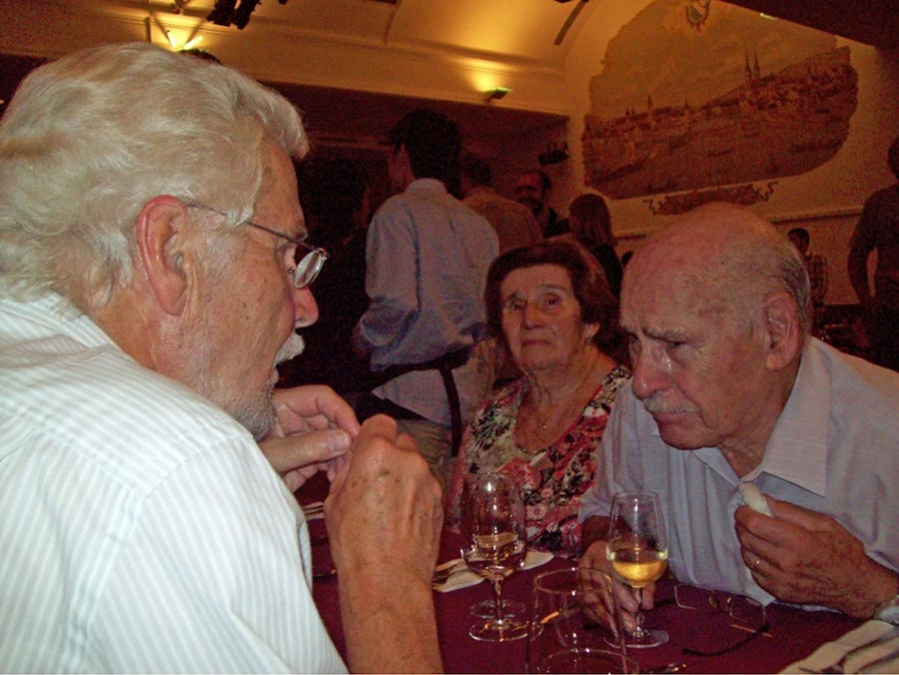
Sigurd von Boletzky discussing with Hélène and Raymond Enay at the conference dinner of the International Symposium Cephalopods—Present and Past, September 9th, 2014 in Zurich, Switzerland. Photo by J.N

**Fig. 4:**
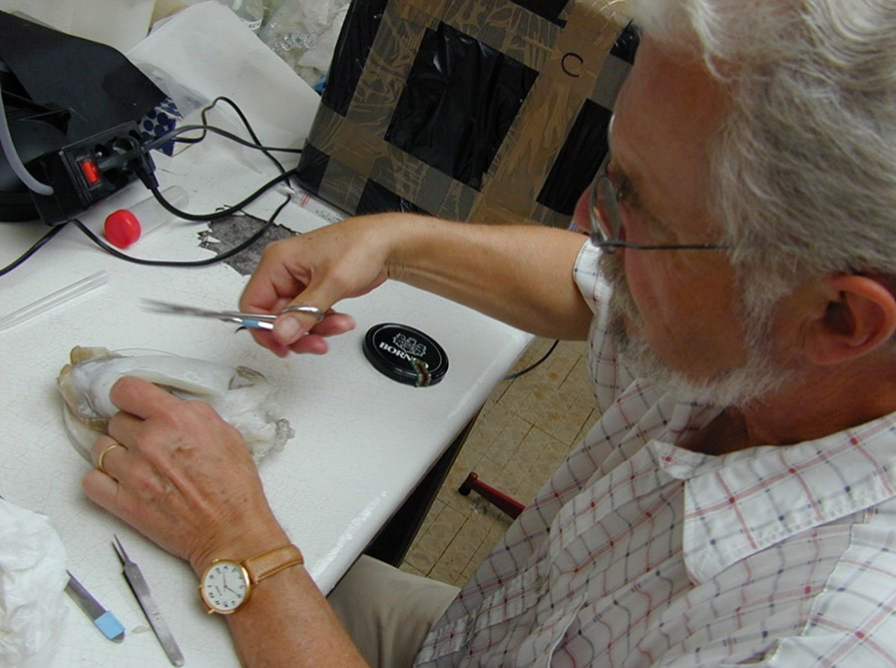
Sigurd von Boletzky in Banyuls, explaining the internal organization of the cuttlefish *Sepia officinalis*

Sigurd was always keen to bring neontologists and palaeontologists together. In 2002, he organized together with Helmut Keupp and his phD student Kerstin Warnke the first International Symposium Coleoid Cephalopods Through Time in Berlin. Moreover, he often attended the International Symposium Cephalopods—Present and Past (Fig. 3), which has a more palaeontological focus but really profits from the presence of neontologists. At the international “Cephalopods—Present and Past” symposium, in Dijon, in 2010, he was awarded a Lifetime Achievement Award. Last but not least, he organized several European symposia on coleoid cephalopods (Fig. 4).

Within the CIESM (Mediterranean Science Commission), Sigurd created, at the end of the 1970s, the ‘Groupe de travail sur les Céphalopodes’, which operated in the Mediterranean area and organized various meetings. The first was held in the Laboratoire Arago in Banyuls in 1981, and was followed by those of Barcelona, Mola di Bari, Creta, Mazara del Vallo, Florence. In this way, Boletzky created a network of mostly young cephalopod researchers—French, Spanish, Italian, German, Greek, Portuguese, Tunisian—who exchanged ideas and experiences essential for their development under his scientific patronage. Some of these meetings were dedicated to specific themes such as “Mediterranean Sepiolidae” (Boletzky, 1995) or the even more international meeting at Mazara on “Squid age determination using statoliths” (Jereb et al., 1991) or the workshop on “Cephalopod parasites” at Banyuls (Pascual et al., 1996).

## Conclusion

Sigurd von Boletzky was one of the leading researchers in cephalopod development and evolution. With his activities, his excitement, and his warm-hearted character, he united biologists and palaeontologists. He will be dearly missed by the community and we will all keep fond memories of our great encounters.

## Data Availability

All sources of information are provided in the text. No fossil or zoological materials were used.
